# Comparison of survivals between sublobar resection and lobar resection for patients with clinical stage I non‐small cell lung cancer and interstitial lung disease: a propensity score matching analysis

**DOI:** 10.1111/1759-7714.15418

**Published:** 2024-09-09

**Authors:** Ryohei Matsushima, Kosuke Fujino, Yamato Motooka, Hiroyuki Yamada, Chika Shirakami, Yusuke Shinchi, Hironobu Osumi, Tatsuya Yamada, Kentaro Yoshimoto, Koei Ikeda, Ichiro Kubota, Makoto Suzuki

**Affiliations:** ^1^ Department of Thoracic Surgery Kumamoto University Hospital Kumamoto Japan; ^2^ Department of Thoracic Surgery National Hospital Organization Minamikyushu Hospital Kagoshima Japan

**Keywords:** interstitial lung disease, lobar resection, lobectomy, non‐small cell lung cancer, sublobar resection

## Abstract

**Background:**

Patients with early‐stage lung cancer and interstitial lung disease have a poorer prognosis than those without interstitial lung disease. This study aimed to compare the long‐term outcomes of lobar and sublobar resections in these patients.

**Methods:**

We retrospectively analyzed 138 consecutive patients with clinical stage I non‐small cell lung cancer and interstitial lung disease who underwent surgical treatment at two institutions between January 2010 and December 2020. Propensity score matching analysis was performed to adjust for baseline characteristics.

**Results:**

Thirty‐six patients underwent sublobar resection and 102 underwent lobar resection. The median follow‐up was 45.7 months. In all patients, 5‐year overall survival (OS) rates were 33.2% and 73.2%, and 5‐year recurrence‐free survival (RFS) rates were 24.2% and 60.1% in the sublobar and lobar resection groups, respectively (*p* < 0.01, <0.01). Death due to lung cancer and locoregional recurrence were significantly more frequent in the sublobar resection group than in the lobar resection group (*p* = 0.034, <0.01, respectively). On propensity score matching analysis, the 5‐year OS rates of the 19 matched pairs were 46.3% and 73.2%, and the RFS rates were 31.6% and 67.6% in the sublobar and lobar resection groups, respectively (*p* = 0.036, <0.01). The Cox proportional hazards model demonstrated a significant association between lobar resection and improved survival (*p* = 0.047).

**Conclusion:**

The patients in the lobar resection group had better survival rates than those in the sublobar resection group. In terms of long‐term prognosis, deliberately limited surgery may not be necessary for patients who tolerate lobectomy.

## Graphical abstract

We retrospectively investigated the long‐term postoperative outcomes of patients with clinical stage I NSCLC and ILD using propensity score matching. In the result, overall survival and recurrence‐free survival were better in the lobar resection group than in the sublobar resection group.

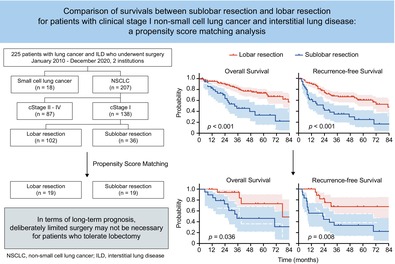

## INTRODUCTION

Interstitial lung disease (ILD) is a specific group of diseases that causes fibrotic scarring of the lungs. Patients with ILD are well known to show a high probability of developing lung cancer.^1^, ^2^, ^3^ Several studies have reported that patients with lung cancer and ILD, particularly idiopathic pulmonary fibrosis (IPF), have a poorer prognosis than patients with lung cancer without ILD.^4^, ^5^, ^6^ Among patients with pathological stage I lung cancer and those with pathological stage I lung cancer with ILD, the 5‐year overall survival rates are reportedly 80%–90% and 42%–59%, respectively.^7^, ^8^ Factors that worsen the prognosis of patients with lung cancer and ILD include the progression of acute exacerbation (AE) of ILD, faster progression of cancer, and limited treatment options.^1^, ^2^, ^4^, ^9^

Lobar resection is the standard surgery for patients with early‐stage non‐small cell lung cancer (NSCLC), but increases the risk of ILD exacerbation.^10^ Several studies have shown that sublobar resections, such as wedge resection or segmentectomy, provide survival rates comparable to those of lobar resection in patients with early‐stage NSCLC.^11^, ^12^ Sublobar resection has been reported to reduce the postoperative exacerbation of ILD and possibly improve survival rates in patients with ILD; thus, sublobar resection could be considered for these patients.^10^, ^13^ However, clear criteria have yet to be defined in terms of surgical strategy for early‐stage NSCLC with ILD in clinical practice.

In the present study, using propensity score matching, we retrospectively investigated the long‐term outcomes and various prognostic factors of patients with clinical stage I NSCLC and ILD who underwent surgery at two institutions.

## METHODS

### Ethical statement

The study was conducted in accordance with the ethical standards of the Declaration of Helsinki and was approved by the review boards of each participating institution (approval dates and numbers: Kumamoto University Hospital, April 1, 2016, genome 402; National Hospital Organization Minamikyushu Hospital, June 14, 2021, 20210614). Informed consent was not required from the individual patients because of the retrospective study design and anonymization of individual data.

### Patients

We retrospectively investigated the clinical data of 138 consecutive patients with clinical stage I NSCLC and ILD who underwent surgical resection between January 2010 and December 2020 at either Kumamoto University Hospital (89 patients) or the National Hospital Organization Minamikyushu Hospital (49 patients). Multiple primary lung cancers, metastatic lung cancers, and surgical biopsies for diagnosis purposes were excluded from the analysis.

Patients were classified into two groups according to the surgical procedure. The sublobar resection group included patients who underwent wedge resection and segmental resection, whereas the lobectomy group included patients who underwent lobectomy. During wedge resection, an endostapler was used to resect the tumor and secure a resection margin that was at least equal to the tumor size. All patients achieved complete tumor resection and R1 cases were excluded from the analysis. Systematic dissection of the hilar or mediastinal lymph nodes was performed for lobectomy and segmental resection but not for wedge resection. However, in patients with possible preoperative lymph node metastases, rapid intraoperative pathology was performed to confirm that the hilar or mediastinal lymph nodes were negative for metastases. The surgical technique was determined at a surgical conference with specialists at each institution. Lobectomy was performed whenever possible, even in the presence of risk factors, on the basis of a comprehensive evaluation of age, sex, respiratory function, history of ILD, and serum KL‐6 levels. No pre‐ or postoperative chemotherapy or radiation therapy was administered.

The primary outcome was overall survival (OS) and the secondary outcomes were independent prognostic factors for OS and recurrence‐free survival (RFS).

### Clinical assessment

Clinical staging was performed according to the eighth TNM classification of the Union for International Cancer Control.^8^ Cases from 2010 to 2017 when the seventh edition series was used were reclassified according to the eighth edition. Tumors, lymph nodes, and distant metastases were assessed using high‐resolution computed tomography (HRCT), positron emission tomography/computed tomography (PET/CT), bone scintigraphy, and brain magnetic resonance imaging. In cases where HRCT or PET/CT showed enlargement (>1 cm) of the mediastinal or hilar lymph nodes or a high accumulation of maximum standardized uptake values (>1.5) in the lymph nodes, endobronchial ultrasound transbronchial needle aspiration, lymph node dissection, or sampling during surgery was performed. ILD was diagnosed according to the American Thoracic Society and European Respiratory Society criteria.^14^, ^15^ HRCT was employed for radiographic validation of a usual interstitial pneumonia (UIP) pattern, which is characterized by the presence of subpleural‐ and basal‐predominant reticular abnormality and honeycomb appearance with or without traction bronchiectasis.^15^ Patients without the presence of a honeycomb appearance were categorized as having a non‐UIP pattern. ILD was diagnosed by surgical and medical oncologists, radiologists, and pathologists. The criteria for postoperative AE of ILD were^1^: acute respiratory deterioration developing within 30 days after pulmonary resection,^2^ presence of a new interstitial shadow on HRCT,^3^ decrease in arterial oxygen tension of more than 10 mmHg from the previous level, and^4^ absence of alternative causes, such as lung infection or heart failure.^14^, ^16^

Medical records included patient characteristics such as age, sex, and pulmonary function, including percentage predicted vital capacity (%VC), percentage predicted diffusing capacity of the lung for carbon monoxide (%DLCO), and serum KL‐6 levels. The Brinkman Index (BI), which represents the number of cigarettes smoked per day multiplied by the number of years of smoking, was used to validate smoking history. Surfactant protein‐D was not assessed because of lack of data. We also collected data on histological characteristics, postoperative mortality, and causes of death. A designation of “peripheral tumor” reflected a tumor location in the outer one‐third of the lung field, while a “proximal tumor” reflected a location in the inner two‐thirds.

After surgery, patients were followed up regularly with physical examinations, chest radiography, and computed tomography (CT) every 6 months over the first 2 years and annually thereafter. Locoregional recurrence was defined as the evidence of a tumor within the same lobe, hilum, or mediastinal lymph node. Distant recurrence was defined as evidence of tumor within another lobe, pleura, pleural space, and bone, including ribs, or elsewhere outside the hemithorax.

### Propensity score matching analysis

To balance potential confounders, we matched the patients in both groups using a propensity score‐matching technique.^17^ Propensity scores were generated using a logistic regression model and the categories in Table 1 were entered as covariates. Cases from the two surgical resection groups were matched 1:1 based on the propensity scores using a nearest neighbor with caliper algorithm.

**TABLE 1:** Patient characteristics.

Categories	Sublobar resection (*n* = 36)	Lobar resection (*n* = 102)	*p*‐value
Age (year)	72.9 ± 7.64	72.3 ± 6.87	0.660
Sex			
Male/female	30/6	82/20	0.808
Brinkman index	1054 ± 743	1011 ± 651	0.680
%VC (%)	91.7 ± 16.3	101.1 ± 17.8	0.007
%DLCO (%)	68.5 ± 24.3	94.0 ± 46.3	<0.001
KL‐6 (U/mL)	685.9 ± 348	534.4 ± 312	0.009
Preoperative medication			
Steroid +/−	6/30	3/99	0.010
Pirfenidone +/−	3/33	8/94	1.000
Radiological pattern			
UIP/non‐UIP	10/26	24/78	0.655
Surgical approach			
VATS/thoracotomy	33/3	88/14	0.559
Tumor location (lobe)			
Upper/middle/lower	12/1/23	34/3/65	1.000
Tumor distribution			
Peripheral/proximal	32/4	70/32	0.017
Clinical stage			
IA1/IA2/IA3/IB	3/17/12/4	7/39/30/26	0.325
Histology			
Ad/Sq/others	12/21/3	53/37/12	0.081
Visceral pleural invasion			
+/−	10/26	33/69	0.679
Surgical procedure			
W/S/L	31/5/0	0/0/102	

*Note*: Categoric data are expressed as number, and continuous data as mean ± standard deviation.

Abbreviations: %DLCO, percentage predicted diffusing capacity of the lung for carbon monoxide; %VC, percentage vital capacity; Ad, adenocarcinoma; KL‐6, Krebs von den Lungen‐6; L, lobectomy; S, segmentectomy; Sq, squamous cell carcinoma; UIP, usual interstitial pneumonia; VATS, video‐assisted thoracoscopic surgery; W, wedge resection.

### Statistical analysis

All descriptive statistics are expressed as numbers or medians based on the Shapiro‐Wilk test, which was used to examine the normality of the data distributions. The Chi‐square or Fisher's exact tests were used to compare the frequencies of categorical variables. Student's *t* test or the Mann‐Whitney U test was used to analyze continuous variables after performing the Kolmogorov‐Smirnov test or Shapiro‐Wilk test for normality of the data. OS was defined as the time from surgery to death from any cause or the last follow‐up. RFS was defined as the time from surgery to the first diagnosis of recurrence, death, or the last follow‐up. Survival rates were estimated using the Kaplan‐Meier method and compared using the log‐rank test. Cox proportional hazards regression analysis was used to estimate the prognostic factors. Multivariate analysis added seemingly relevant explanatory variables to the univariate analysis to identify the independent factors. Statistical analyses were performed by a specialist (Satista Co., Ltd., Tokyo, Japan) and the Statistical Package for the Social Sciences (SPSS version 24; IBM Japan, Ltd., Tokyo, Japan). All reported *p*‐values were two‐sided, and values less than 0.05 were considered statistically significant.

## RESULTS

### Patient characteristics

The clinical characteristics of the patients are summarized in Table 1. A total of 36 patients underwent sublobar resection (wedge resection, 31 patients; segmentectomy, 5 patients) and 102 patients underwent lobar resection. The two groups were equivalent in age, sex, BI, and radiological UIP patterns. In terms of pulmonary function testing, %VC and %DLCO were significantly lower in the sublobar resection group (91.7% vs. 101.1% and 68.5% vs. 94.0%, *p* = 0.007 and <0.001, respectively). KL‐6 was higher in the sublobar resection group. Steroid use and peripheral tumors were significantly more frequent in the sublobar group than in the lobar group. The clinical stage distribution for the entire cohort was as follows: stage IA1, 10 (7.2%); stage IA2, 56 (40.5%); stage IA3, 42 (30.4%); and stage IB, 30 (21.7 %) patients. Eight patients were defined as having stage IB disease because of suspected visceral pleural invasion, although the tumors were less than 3 cm in diameter. The number of patients with visceral pleural invasion did not differ between the two groups.

### Postoperative death and cancer recurrence

Most deaths were attributed to lung cancer or respiratory failure (Table 2). Deaths due to lung cancer were significantly more frequent in the sublobar resection group (27.8% vs. 11.8%, *p* = 0.034). Conversely, the postoperative AE of ILD was observed only in the lobar resection group. AE of ILD developed in five patients in the lobar resection group: two patients died 30 and 144 days after surgery, three patients recovered with steroid pulse therapy. The other respiratory failures listed in Table 2 included bacterial pneumonia, pulmonary mucormycosis, pulmonary hypertension, and chronic exacerbation of ILD (exacerbation of ILD occurring after 30 days postoperatively due to any cause). Deaths due to other causes were significantly more frequent in the sublobar resection group (*p* = 0.02). These deaths were due to acute peritonitis, acute pancreatitis, ureteral cancer, leukemia, multiple trauma, or unknown causes.

**TABLE 2:** Details of death and recurrence.

	Sublobar resection (*n* = 36)	Lobar resection (*n* = 102)	*p*‐value
Cause of death			
Lung cancer	10 (27.8%)	12 (11.8%)	0.034
Respiratory failure	2 (5.6%)	12 (11.8%)	0.357
Postoperative acute exacerbation of ILD	0 (0.0%)	2 (2.0%)	1.000
Other respiratory failure	2 (5.6%)	10 (9.8%)	0.731
Others	6 (16.7%)	4 (3.9%)	0.02
Site of recurrence			
Locoregional	8 (22.2%)	6 (5.9%)	0.009
Distant	8 (22.2%)	18 (17.7%)	0.621

Abbreviation: ILD, interstitial lung disease.

The locoregional recurrence rate was significantly higher in the sublobar resection group than that in the lobar resection group (22.2% vs. 5.9%, *p* = 0.009) (Table 2), whereas no differences were observed in the distant recurrence rate (22.2% vs. 17.7%, *p* = 0.621). Common sites of recurrence were the surgical margins for locoregional recurrences, and the bones and contralateral lungs for distant recurrences.

### Survival

**FIGURE 1:**
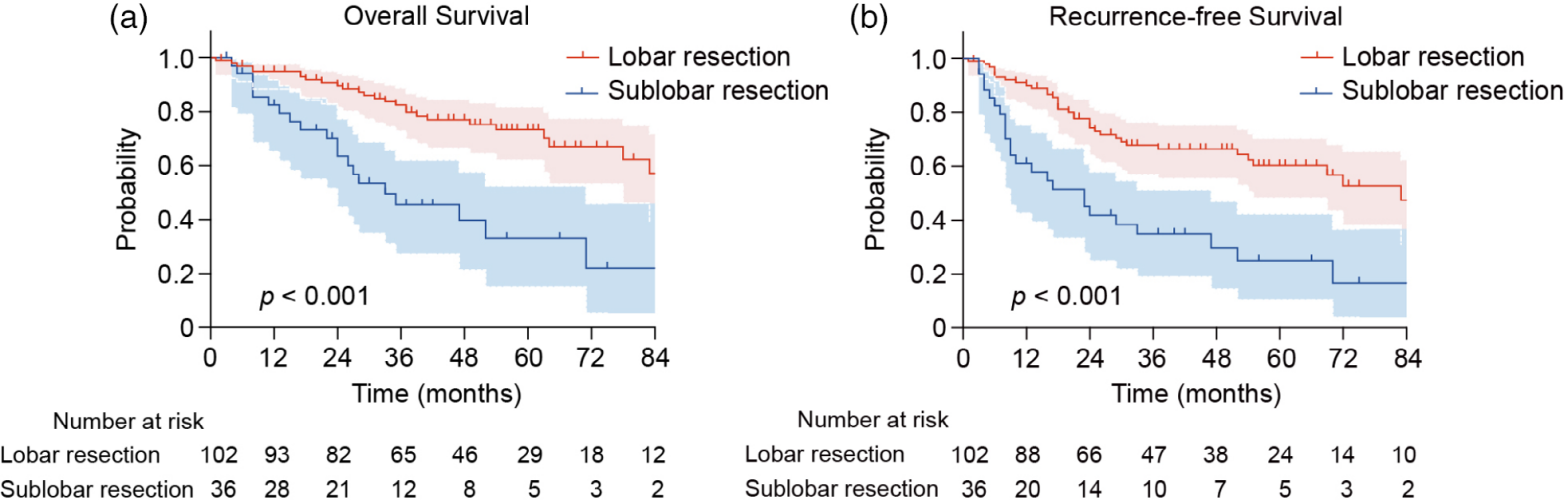
Overall survival (a) and recurrence‐free survival (b) of all clinical stage I non‐small cell lung cancer patients with interstitial lung disease.

The median follow‐up durations were 45.7, 48.4 and 35.3 months for the entire cohort, the lobar resection group, and the sublobar resection group, respectively. Three‐ and 5‐year OS rates were 45.5% and 33.2% in the sublobar resection group, and 81.0% and 73.2% in the lobar resection group, respectively (*p* < 0.001) (Table 3). Three‐ and 5‐year RFS rates were 33.8% and 24.2% in the sublobar resection group and 66.1% and 60.1% in the lobar resection group, respectively (*p* < 0.001). Kaplan‐Meier estimates showed significant differences in OS and RFS between the groups (Figure 1a,b). The 30‐ and 90‐day mortality rates were 1.0% in the lobar resection group and 0% in the sublobar resection group (*p* = 1.000).

**TABLE 3:** Outcomes: survival and mortality.

	Sublobar resection (*n* = 36)		Lobar resection (*n* = 102)		*p*‐value
	Proportion	95% CI	Proportion	95% CI	
Overall survival					
3 year	0.455	0.272–0.621	0.81	0.712–0.878	<0.001
5 year	0.332	0.151–0.525	0.732	0.617–0.817	<0.001
Recurrence‐free survival					
3 year	0.338	0.184–0.500	0.661	0.551–0.751	<0.001
5 year	0.242	0.102–0.413	0.601	0.480–0.703	<0.001
Mortality					
30 day	0 (0.0%)		1 (1.0%)		1.000
90 day	0 (0.0%)		1 (1.0%)		1.000

Abbreviation: CI, confidence interval.

### Univariate and multivariate analysis of prognostic factors

Univariate Cox regression analysis for OS showed that age, %VC, serum KL‐6 level, UIP pattern, and surgical procedure had *p* < 0.05 (Table 4). Multivariate analysis identified age, KL‐6 levels, and surgical procedure as prognostic factors (*p* < 0.001, = 0.002, = 0.007, respectively). Sublobar resection showed a higher hazard ratio (HR, 2.350; 95% confidence interval [CI]: 1.253–4.433) for OS than did lobar resection.

**TABLE 4:** Univariate and multivariate cox regression analysis for overall survival.

Variables	Univariate			Multivariate		
	Hazard ratio	95% CI	*p*‐value	Hazard ratio	95% CI	*p*‐value
Age (per 1)	1.074	1.029–1.122	0.001	1.095	1.041–1.151	<0.001
Sex				‐		
Male	1.471	0.624–3.468	0.378			
Female	1.000	ref				
Brinkman index (per 1)	1.000	0.997–1.000	0.707	‐		
%VC (per 1)	0.978	0.960–0.995	0.011	0.986	0.967–1.005	0.138
%DLCO (per 1)	0.990	0.978–1.003	0.127	‐		
KL‐6 (per 1)	1.001	1.001–1.002	<0.001	1.002	1.001–1.002	0.002
Radiological diagnosis						
UIP pattern	1.941	1.070–3.523	0.029	1.713	0.903–3.251	0.100
Non‐UIP pattern	1.000	ref		1.000	ref	
Histology				‐		
Adenocarcinoma	1.000	ref				
Squamous cell carcinoma	1.847	0.972–3.510	0.061			
Others	2.200	0.941–5.144	0.068			
Surgical procedure						
Sublobar resection	3.318	1.845–5.970	<0.001	2.350	1.253–4.433	0.007
Lobar resection	1.000	ref		1.000	ref	

Abbreviations: %DLCO, percentage predicted diffusing capacity of the lung for carbon monoxide; %VC, percentage vital capacity; CI, confidence interval; KL‐6, Krebs von den Lungen‐6; UIP, usual interstitial pneumonia.

### Propensity score matching analysis

**FIGURE 2:**
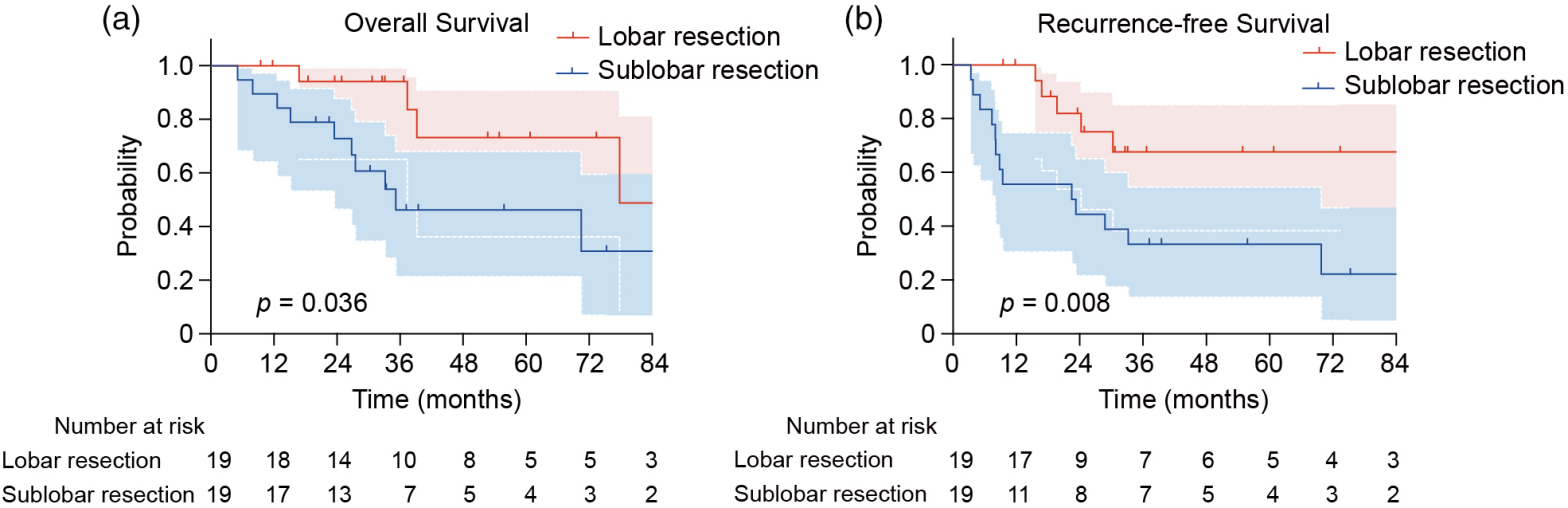
Overall survival (a) and recurrence‐free survival (b) between propensity matched patients undergoing lobar resection or sublobar resection.

Propensity score matching yielded 19 matched pairs that showed similar characteristics between the two groups (Table 5). Differences in %VC, %DLCO, KL‐6, steroid use, and tumor location (Table 1) were no longer observed after matching. The 5‐year OS and RFS rates were significantly higher in the lobar resection group than in the sublobar resection group (Table 6; Figure 2). Cox regression analysis demonstrated a significant association between OS and surgical procedure (Table 7).

**TABLE 5:** Patient characteristics after propensity score matching.

Categories	Sublobar resection (*n* = 19)	Lobar resection (*n* = 19)	*p*‐value
Age (y)	71.6 ± 6.89	71.2 ± 7.35	0.839
Sex			
Male/female	17/2	17/2	1.000
Brinkman index	1122 ± 663	967.4 ± 516	0.425
%VC (%)	92.9 ± 17.4	94.0 ± 14.57	0.827
%DLCO (%)	72.7 ± 25.6	75.0 ± 18.7	0.748
KL‐6 (U/mL)	708.9 ± 312	717.0 ± 428	0.708
Preoperative medication			
Steroid +/−	2/17	1/18	1.000
Pirfenidone +/−	1/18	1/18	1.000
Radiological pattern			
UIP/non‐UIP	6/13	5/14	1.000
Surgical approach			
VATS/thoracotomy	18/1	18/1	1.000
Tumor location (lobe)			
Upper/middle/lower	8/1/10	9/0/10	1.000
Tumor distribution			
Peripheral/proximal	16/3	13/6	0.447
Clinical stage			
IA1/IA2/IA3/IB	1/8/6/4	2/8/6/3	1.000
Histology			
Ad/Sq/others	8/10/1	7/11/1	1.000
Visceral pleural invasion			
+/−	6/13	4/15	0.714
Surgical procedure			
W/S/L	14/5/0	0/0/19	

*Note*: Categorical data are expressed as numbers, and continuous data as mean ± standard deviation.

Abbreviations: %DLCO, percentage predicted diffusing capacity of the lung for carbon monoxide; %VC, percentage vital capacity; Ad, adenocarcinoma; KL‐6, Krebs von den Lungen‐6; L, lobectomy; S, segmentectomy; Sq, squamous cell carcinoma; UIP, usual interstitial pneumonia; VATS, video‐assisted thoracoscopic surgery; W, wedge resection.

**TABLE 6:** Five‐year survival after propensity score matching.

	Sublobar resection (*n* = 19)		Lobar resection (*n* = 19)		*p*‐value
	Proportion	95% CI	Proportion	95% CI	
Overall survival	0.463	0.213–0.681	0.732	0.361–0.909	0.036
Recurrence‐free survival	0.316	0.129–0.522	0.676	0.383–0.852	0.008

Abbreviation: CI, confidence interval.

**TABLE 7:** Cox regression analysis for overall survival after propensity score matching.

Variables	Hazard ratio	95% CI	*p*‐value
Age (per 1)	1.068	0.986–1.158	0.105
Sex			
Male	0.693	0.085–5.645	0.732
Female	1.000	ref	
Brinkman Index (per 1)	1.000	0.999–1.001	0.818
%VC (per 1)	0.993	0.960–1.027	0.668
%DLCO (per 1)	0.983	0.960–1.007	0.162
KL‐6 (per 1)	1.001	0.999–1.002	0.268
Radiologic diagnosis			
UIP pattern	1.617	0.525–4.986	0.403
Non‐UIP pattern	1.000	ref	
Histology			
Adenocarcinoma	1.000	ref	
Squamous cell carcinoma	0.530	0.167–1.681	0.281
Others	2.492	0.494–12.57	0.269
Surgical procedure			
Sublobar resection	3.269	1.013–10.55	0.047
Lobar resection	1.000	ref	

Abbreviations: CI, confidence interval; %VC, percentage vital capacity; %DLCO, percentage predicted diffusing capacity of the lung for carbon monoxide; KL‐6, Krebs von den Lungen‐6; UIP, usual interstitial pneumonia.

## DISCUSSION

Surgical treatment for patients with lung cancer and ILD should be carefully considered because of the high perioperative mortality rate. Lobar resection is the standard treatment for early‐stage lung cancer, whereas sublobar resection such as segmentectomy or wedge resection can be performed in patients with small peripheral lung cancers or in high‐risk operable patients.

In the present study, the survival rate was higher in the lobar resection group than that in the sublobar resection group among patients with clinical stage I NSCLC and ILD. In addition, the Cox regression model suggested that the surgical procedure was an important factor influencing prognosis. These results indicate that lobar resection for patients with early‐stage NSCLC and ILD is practical and effective in terms of long‐term prognosis. A retrospective, large‐scale study showed a similar trend in that among patients with stage IA lung cancer and ILD, the 5‐year OS rates with wedge resection, segmentectomy, and lobectomy were 29.2%, 60.0%, and 68.6%, respectively.^7^ That study reported cancer recurrence as the main cause of death and highlighted the importance of oncological complete resection. Similarly, in this study, sublobar resection was more likely to result in cancer recurrence and lobar resection was likely to cause respiratory failure. The high malignancy rate of lung cancer in patients with ILD makes oncological control particularly important. Tsutani et al. reported that among patients with stage IA NSCLC and ILD with UIP patterns, the 3‐year OS rate tended to be better in the sublobar resection group than in the lobar resection group, although the difference was not statistically significant (81.0% vs. 50.5%, *p* = 0.11).^13^ Joo et al. described similar OS rates in patients with lung cancer and IPF between the lobar and sublobar resection groups.^18^ Our cohort included not only patients with UIP/IPF but also patients with other types of ILD; therefore, further subgroup analysis for each category of ILD is necessary. Taken together, it is necessary to select a surgical procedure that considers the balance between oncological control and pulmonary function according to the individual patient background. Importantly, this study showed that patients eligible for lobar resection may have a relatively good long‐term prognosis. Lobar resection is feasible and effective with careful postoperative management and cautious selection of surgical procedures at conferences with various specialists.

Three‐ and 5‐year RFS rates in the sublobar and lobar resection groups were 33.8% and 66.1% and 24.2% and 60.1%, respectively (*p* < 0.001). These results are broadly consistent with previous reports of 49.2% (95% CI: 39.4–61.4%) for the 5‐year RFS rate^19^ and 21.6% versus 69.6% in the 5‐year RFS rate in sublobar and lobar resection groups, respectively.^18^ Some reports have shown that lung cancer with ILD is associated with an increased risk of cancer recurrence compared with the general lung cancer population.^5^, ^7^, ^18^, ^19^ Song et al. also reported a high rate of local recurrence for lung cancer with IPF after surgery.^19^ In our study, the locoregional recurrence rate was significantly higher in the sublobar resection group (22.2%, *p* = 0.009), despite having a resection margin of at least the tumor size. Local control is important because additional treatment options for patients with ILD remain limited, contributing to the significant difference in OS observed in the present study.

Perioperative complications are critical factors that need to be considered. Although no significant difference was noted, two deaths due to AE of ILD were observed after surgery in the lobar resection group. The Kaplan‐Meier curve for OS displayed a slight crossing a few months after surgery. As some reports have mentioned, lobar resection in patients with ILD can lead to poor short‐term survival owing to the postoperative AE of ILD and other severe complications.^13^, ^20^, ^21^ Sato et al. identified lobar resection as one of the seven risk factors for postoperative AE of ILD.^10^ The risk scoring system proposed by the Japanese Association for Chest Surgery is useful in assessing the preoperative risk of AE of ILD.^22^ Pirfenidone treatment, which was demonstrated to suppress the progression of IPF in a phase III trial, offers the possibility of preventing postoperative AE of ILD but remains under investigation.^23^, ^24^ Perioperative use of pirfenidone might make lobectomy a better option for high‐risk lung cancer patients with ILD.

This study had some limitations that must be considered. First, this was a retrospective, two‐institution, cohort study. Although we performed propensity score matching to reduce selection bias, it was not possible to determine the conclusive superiority or inferiority of the surgical procedures. Further prospective multicenter studies are required to validate our findings. Second, only a small number of patients underwent sublobar resection. In the propensity score matching analysis, there were only 19 matched pairs. More cases need to be accumulated to improve reproducibility. Third, a systematic dissection of the lymph nodes was not performed for wedge resection. Although we performed intraoperative rapid biopsy of the sampled lymph nodes, we consider that it is still a non‐negligible bias that can worsen the survival of the sublobar resection group.

In conclusion, in patients with early‐stage NSCLC and ILD, lobar resection offers better survival than sublobar resection. In terms of long‐term prognosis, deliberately limited surgery might not be necessary for patients who tolerate lobectomy. Although reduction surgery has been widely performed in patients with early‐stage lung cancer in recent years, we believe that sublobar resection should be carefully selected based on sufficient preoperative evaluation, especially for lung cancer patients with ILD.

## AUTHOR CONTRIBUTIONS

Conception and design: Ryohei Matsushima. Administrative support: Ichiro Kubota, Makoto Suzuki. Provision of study materials or patients: Ryohei Matsushima, Kosuke Fujino, Kentaro Yoshimoto, Koei Ikeda. Collection and assembly of data: Ryohei Matsushima, Kosuke Fujino, Hiroyuki Yamada, Chika Shirakami, Yusuke Shinchi, Tatsuya Yamada, Yamato Motooka. Data analysis and interpretation: Ryohei Matsushima, Hironobu Osumi, Kosuke Fujino. Manuscript writing: All authors. Final approval of manuscript: All authors.

## CONFLICT OF INTEREST STATEMENT

The authors have no conflicts of interest to declare.

## Data Availability

All the data are available within the article.
